# The role of mast cells on angiogenesis in oral squamous cell carcinoma

**DOI:** 10.4317/medoral.17395

**Published:** 2011-12-06

**Authors:** Manpreet Kalra, Nirmala Rao, Kanwardeep Nanda, Farzan Rehman, KL. Girish, Shoaib Tippu, Asit Arora

**Affiliations:** 1 Associate Professor, Department of Oral Pathology and Microbiology, SGT Dental College, Gurgaon, India; 2 Professor, Department of Oral Pathology and Microbiology, Manipal College of Dental Sciences, Manipal, India; 3 Senior lecturer, Department of Oral Pathology and Microbiology, SGT Dental College, Gurgaon, India; 4 Department of Oral Pathology and Microbiology, Jaipur Dental College, Jaipur, India; 5 Senior Resident, Department of Surgery, G B Pant Hospital, New Delhi, India

## Abstract

Objective: Angiogenesis or neovascularization has long been known to aid in progression and metastasis of malignant tumors. Tumor angiogenesis is a complex event mediated by angiogenic factors released from cancer cells and or by host immune cells. Mast cells may induce tumor progression and potentiate metastasis by stimulating angiogenesis. The purpose of the present study was to validate topographic distribution of micro vessel density (MVD) and mast cell density (MCD) and help to elucidate the possible role of mast cells in tumor angiogenesis and correlating this with advanced disease parameters. Study Design: MVD and MCD were investigated in tumor specimens from 30 patients diagnosed with different histologic grades of oral squamous cell carcinoma (OSCC). Intratumor vessels were stained with collagen Type IV antibody and mast cells with Toluidine blue before being measured by light microscopy. Results: There was a significant correlation between MVD and disease progression and number of blood vessels increased from well to poorly differentiated OSCC where as MCD decreased. Conclusions: These findings suggest that angiogenesis indeed occur in OSCC and might be used as an index to inflect the aggression of the disease however mast cells make up only a part of complex process of angiogenesis along with other factors secreted by tumor.

** Key words:**Angiogenesis, mast cells, oral squamous cell carcinoma, progression, metastasis.

## Introduction

Oral Squamous Cell Carcinoma is the sixth most common cancer in the world and is one of the leading causes of death in India. Sixty percent of oral cancers are well advanced by the time they are detected and despite innovation being made in surgery, radiation and chemotherapy, the long term survival rate remains to be less than 50% (1). By the time oral cancer is diagnosed, most individuals have localized or regional disease (37% localized; 43% regional; 10% distant and 10% unstaged). Five year survival rates for all oral cancers cases are 79% for those with localized disease, 42% for regional disease and 19% for disease with distant metastases. Development of oral cancer proceeds through discrete molecular genetic changes that are acquired from loss of genomic integrity following continued exposure to environmental risk factors (2). These genetic changes generate concomitant phenotypic changes in the tumor cells allowing them to continually survive, spread out and having an ability to invade surrounding tissues and metastasize (3,4). 

Angiogenesis – the growth of new blood vessels from pre-existing ones is a complex phenomenon that is absolutely required for the continued growth and survival of neoplasms (5). The growth of tumors beyond few millimeters depends on the establishment of microcirculation. The fact that tumors are angiogenesis dependent and metastatic cells are only shed after the tumor establishes its microcirculation has been described in melanomas, breast carcinomas and other malignant neoplasms (6). Angiogenesis as a central process in many human malignancies has been proposed to be regulated by a balance between angiogenic stimulators and inhi-bitors (7). The “angiogenic switch” however depends on net balance of positive and negative angiogenic factors in the tumor. Thus, the angiogenic phenotype may result from the production of growth factors, such as FGF-2 and VGEF, by the tumor cells and/or the down-regulation of negative modulators, like TSP-1, in tissues with quiescent vasculature (8). The angiogenic phenotype in a tumor can be triggered by hypoxia resulting from the increasing distance of the growing tumor cells to the capillaries or from the inefficiency of the newly formed vessels. Also, several oncogenes such as V-ras, K-ras, v-raf, src, fos and V-yes (9-11) induce the up-regulation of angiogenic factors like VGEF and increase the production of cytokines and proteolytic enzymes (12).

This complex event in addition to being mediated by angiogenic factors released from cancer cells also has the host contribution. Among the host immune cells, a role has been implicated for mast cells in tumor progression via promoting angiogenesis. The role of mast cells which are reservoirs of angiogenic peptides in neovascularization is not clear. However mast cells products such as histamine, basic fibroblast growth factor and heparin have been shown to promote tumor angiogenesis. Although mast cells (MCs) have been implicated in promoting angiogenesis in some malignant tumors, little is known in oral squamous cell carcinoma (OSCC) (13,14).

Studies on the topographical distribution of microvessel and mast cell hot spots might help elucidate the possible role of mast cells in tumor angiogenesis in OSCC. Thus the present study is focused at examining the microvessel density by employing type IV collagen a component of vessel wall in different histological grades of OSCC and to correlate angiogenesis with disease progression, and further to determine the role of mast cells in tumor angiogenesis. 

## Material and Methods

 -Tissue material

The material for the study included 30 formalin-fixed paraffin-embedded tissue blocks of histologically proven cases of OSCC from the archives of Department of Oral & Maxillofacial Pathology, Manipal College of Dental Sciences, Manipal, between the years 2003 and 2006. Ten blocks of pyogenic granuloma and normal oral mucosa were used as controls. Immunohistochemical analysis using type IV collagen was done to assess the nature and distribution of blood vessels in different histological grades. Special stain Toludine blue for mast cells were carried out in all these cases.

MicroVessel Density (MVD)

The immunohistochemical analysis for blood vessel was performed using streptavidine-biotin-peroxidase technique. Paraffin- embedded tissues were cut into 4-µm- thick sections and taken onto poly -L- Lysine (Sigma Aldrich Chemical Co., St Louis, MO, USA) adhesive oated micro slides. The sections were brought to water, treated with xylene three times for 5 min and rehydrated with ethanol. Quenching of endogenous peroxidase activity was achieved by immersing the slides in freshly prepared 3% H2O2 in methanol. For antigen retrieval, the sections were digested with 2mg/ml Pepsin in 0.1M HCl at room temperature for 30 mins, and then washed in distilled water. After a brief rinsing in phosphate buffered saline (PBS), sections were treated with 10% goat serum at room temperature for 30min to block any non-specific antigenic sites. The sections were then incubated with primary antibody (anti collagen type IV Sigma Aldrich Chemical Co.) at 1:50 dilution, for 60 minutes in a humid chamber. For reagent control, no primary antibody was added. After washing in PBS sections were incubated with biotinylated secondary antiboby at 1:200 dilutions at room temperature for 30 min (Sigma Aldrich Chemical Co). Following a PBS wash for 5min thrice, sections were incubated with streptavidin-peroxidase conjugate (Sigma Aldrich Chemical Co.), at a concentration of 1:200 for 30 min and detected using 0.05% 3,3-diaminobenzidine tetra-hydrochloride (DAB) (Dakocytomation, Glostrup, Denmark). The slides were counter-stained with Meyer’s hematoxylin, subsequent to which sections were dehydrated, cleared and mounted with DPX and cover slipped.

 -Mast Cell Density (MCD)

In each of 30 cases, additional sections from the tissue blocks were taken and were stained with 0.1% aqueous Toluidine blue for observing the Mast cells in the tissue. 

The stain gave a light blue background to the section with mast cells appearing red-purple in color. This allowed easy mapping of stained mast cells. 

 -Evaluation

**Figure 1:**
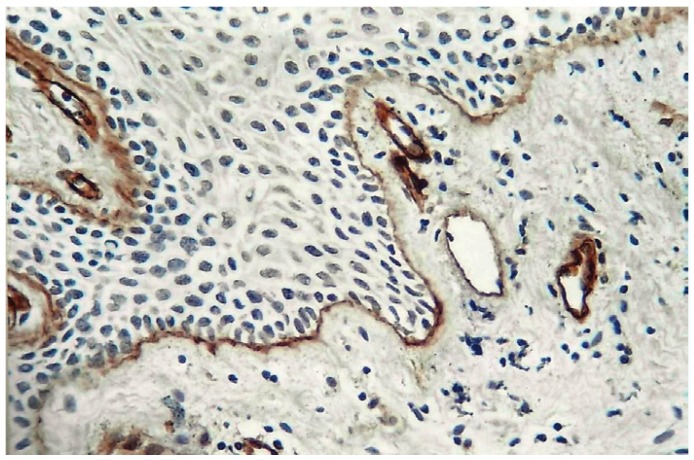
Shows type IV collagen immunopostivity in basement membrane around blood vessels located subepithelialy in normal mucosa (10X).

Evaluation of immune staining was performed by two observers. The initial evaluation was qualitative in order to examine the different expression pattern of the antibody. It was performed in all section. For quantitative analysis of Micro Vessel Density (MVD) the stained sections were screened at low power (4X) to identify the areas of highest vascularisation within the tumor (hot spots). Microvessel counts was performed in four fields at 10X magnification with the use of an ocular grid subdivided into 100 areas in four fields of vision, and for each field the hotspot MVD was noted. The blood vessels were identified by presence of brown stained basement membrane around them (Fig. 1). The vessel lumen, although usually present, was not a criterion used to define a microvessel, and red blood cells were not used to define vessel lumens. Partially identified vessels, which were not totally contained in the field analysed, were not considered in vessel counts. 

Mast cells were identified by their characteristic metachromasia. By using the same counting methods as for MVD, mast cells were counted in hot spots to determinate the Mast Cell Density value. 

 -Statistical Analysis

Microvessel density and Mast cell density was quantitatively assessed by two independent observers to remove any possible bias. Statistical analysis was performed using SPSS statistical software package version 15.0 (SPSS, Chicago). Non- parametric Mann-Whitney test was applied to statistically evaluate two group differences and Kruskal-wallis (ANOVA) test for three group differences. P-values of < 0.05 and < 0.001 were considered to be significant and very highly significant, respectively.

## Results

 -Microvessel Density

**Figure 2:**
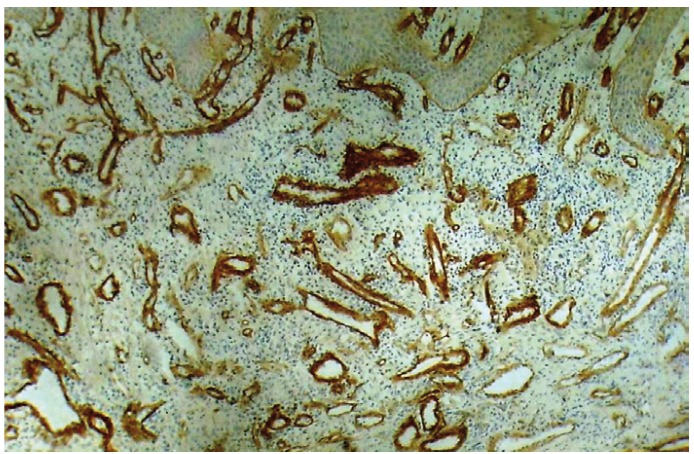
Shows circumferential arrangement of type IV collagen immunopositive blood vessels in Well Differentiated OSCC (25X).

**Figure 3:**
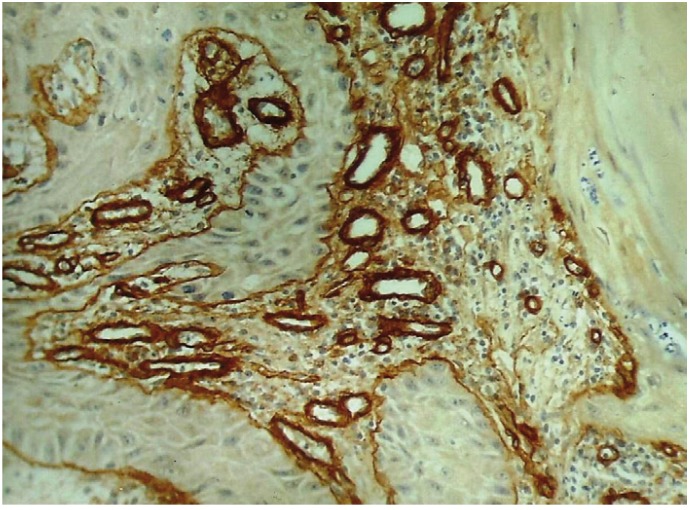
Shows an irregular arrangement blood vessels immunopositive for type IV collagen in Moderately Differentiated OSCC (25X).

**Figure 4:**
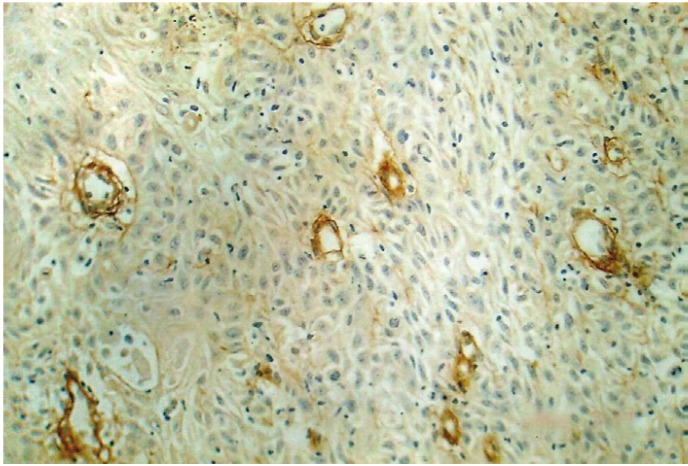
Shows an irregular arrangement of type IV collagen immunopositive blood vessels in Poorly Differentiated OSCC (25X).

It was observed that all the 30 OSCC cases tested for the expression of Collagen IV, showed immunopositivity. Immunohistochemical staining of blood vessels with Collagen IV was within the basement membrane which stained positively. Intratumoral blood vessels concentrated in discrete hotspots both within sheets of tumor cells in carcinomas with a pushing margin and in areas containing leukocyte infiltration in carcinomas with an invasive margin (Fig. 2, 3, 4). For quantitative analysis two observers independently counted the blood vessels within the tumor (hot spots) to minimize the subjective bias.

When the number of blood vessels were evaluated in well, moderately and poorly differentiated OSCCs, the mean number of blood vessels were found to be 51.55, 90.15 and 164.25 respectively. Kruskal-wallis test (ANOVA) was applied on the results to compare the number of blood vessels in different grades of OSCCs, p value was found to be very highly significant (p= 0.001) ( Table 1).

When the number of blood vessels were compared between the different grades of OSCC, well vs moderately differentiated – mean difference of 38.60 vessels was observed; well vs poorly differentiated – mean difference of 112.70 vessels was observed; moderately vs poorly differentiated – mean difference of 74.10 vessels was observed.

**Figure 5:**
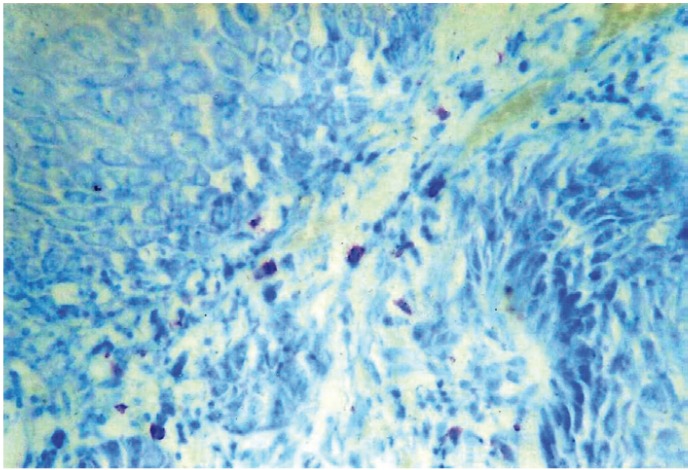
Shows Mast cells positive for toluidine blue in Well Differentiated OSCC (40X).

These inter comparison results were assessed by Mann – Whitney U test. From these observations, it was imperative that p value was very highly significant in all the inter comparisons i.e. well vs moderately differentiated (p = 0.001), well vs poorly differentiated (p=0.001) and moderately vs poorly differentiated (p= 0.001) ( Table 2). Hence, the number of blood vessels increased in transition from well differentiated to moderately differentiated and from moderately differentiated to poorly differentiated OSCC (Fig. 5) ( Table 2).

 -Mast cell Density

**Figure 6:**
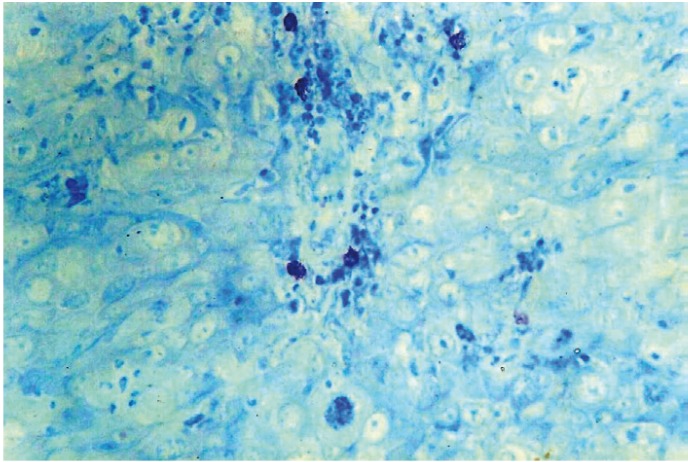
Shows Mast cells positive for Toluidine blue adjacent to tumor cells in OSCC (40X).

**Figure 7:**
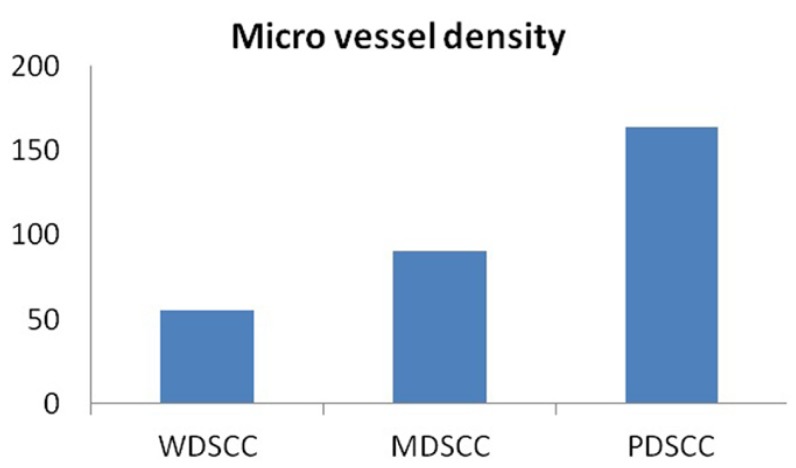
WDSCC: Well differentiated squamous cell carcinoma. MDSCC: Moderately differentiated squamous cell carcinoma. PDSCC: Poorly differentiated squamous cell carcinoma.

By using the same counting methods as for blood vessels, mast cells were counted in hot spots (Fig. 6,7). To eliminate the subjective bias, two observers independently counted the mast cells. When the number of mast cells were evaluated in well, moderately and poorly differentiated OSCCs, the mean number of mast cells were found to be 12.85, 7.62 and 5.05 respectively. Kruskal-wallis test (ANOVA) was applied on the results to compare the number of mast cells in different grades of OSCCs, p value was found to be highly significant (p= 0.010) ( Table 3).

When the number of mast cells was compared between the different grades of OSCC, well vs moderately differentiated – mean difference of 5.225 cells were observed; well vs poorly differentiated – mean difference of 7.800 cells were observed; moderately vs poorly differentiated – mean difference of 2.575 cells was observed.

**Figure 8:**
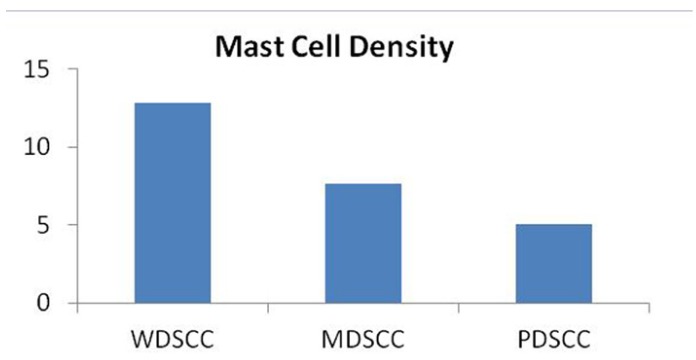
WDSCC: Well differentiated squamous cell carcinoma. MDSCC: Moderately differentiated squamous cell carcinoma PDSCC: Poorly differentiated squamous cell carcinoma.

These inter comparison results when subjected to Mann – Whitney U test, p value was found to be not significant in the inter comparisons between well vs moderately differentiated (p = 0.064) and moderately vs poorly differentiated (p= 0.212). The p value was found to be highly significant in the inter comparison between well vs poorly differentiated (p=0.003) ( Table 4). Hence, the number of mast cells decreased in transition from well differentiated to poorly differentiated OSCC (Fig. 8) ( Table 4). In the present study the number of blood vessels increased from well to poorly differentiated showing an inverse relationship with the differentiation of the tumor. In contrast, the number of mast cells decreased from well differentiated to poorly differentiated OSCC.

**Table 1:**
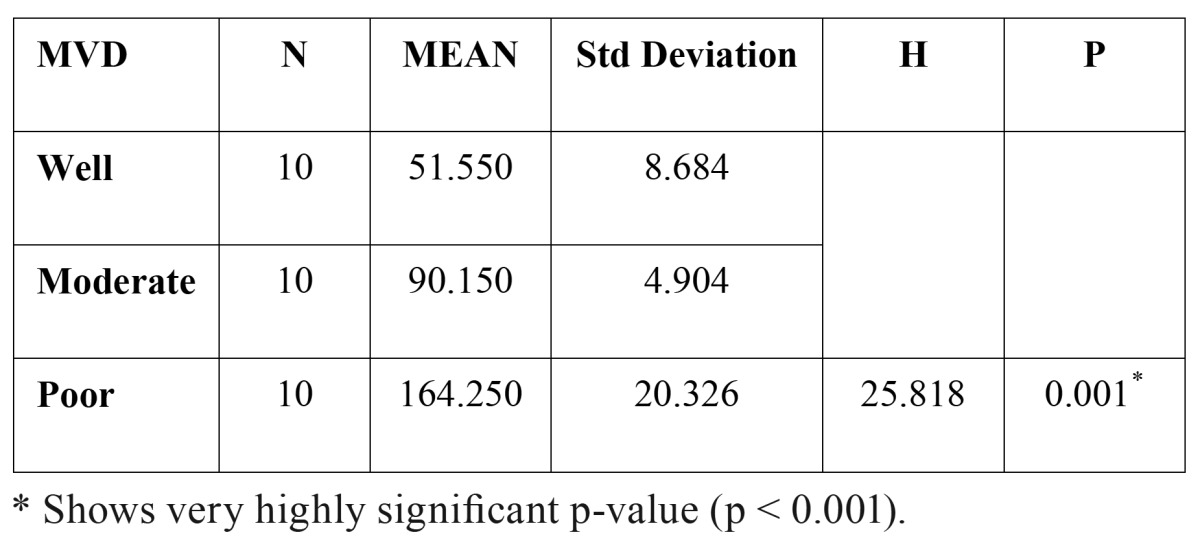
Micro vessel density (MVD) in different grades of OSCC.

**Table 2:**
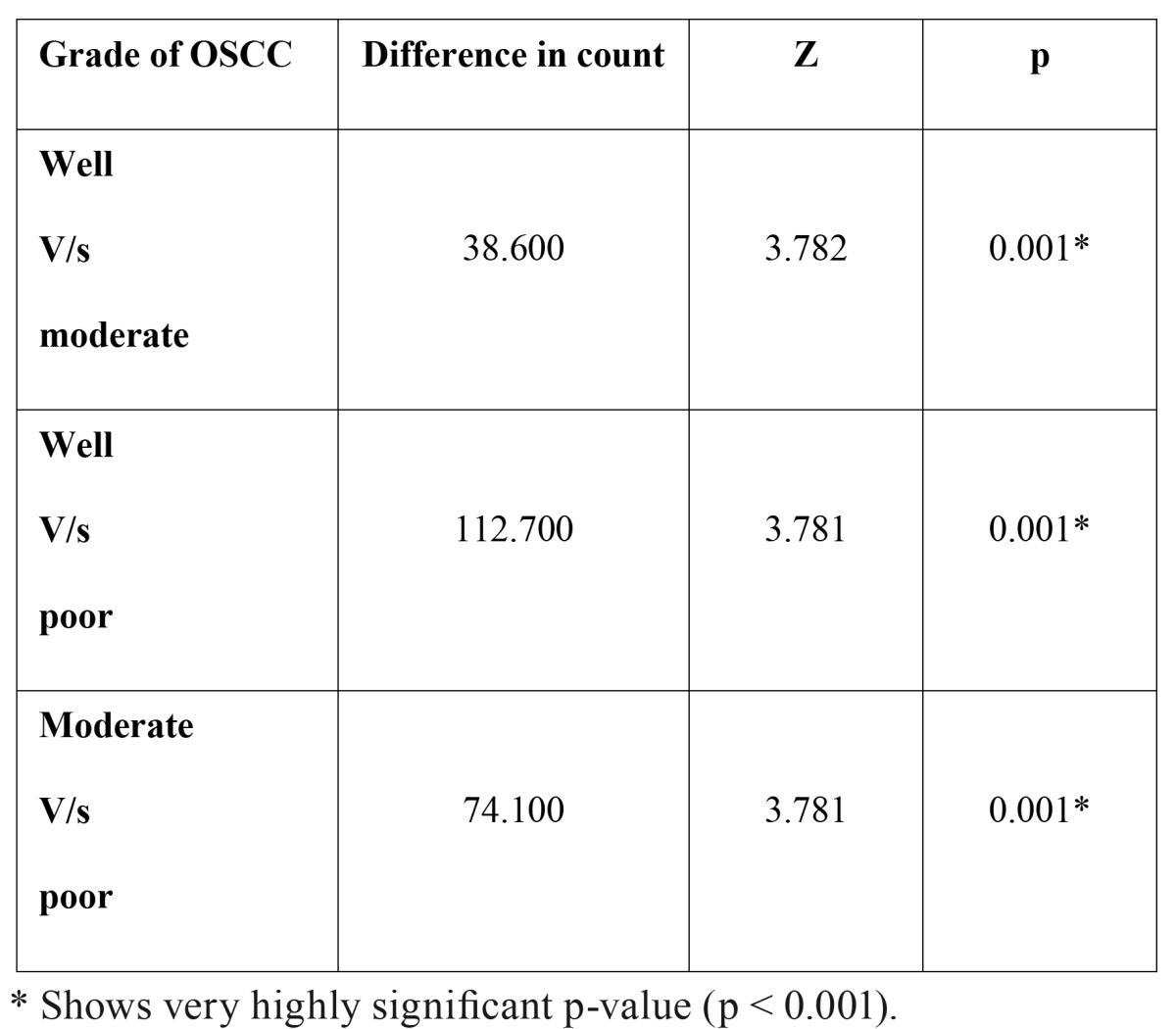
The comparison of micro vessel density (MVD) between different grades of OSCC.

**Table 3:**
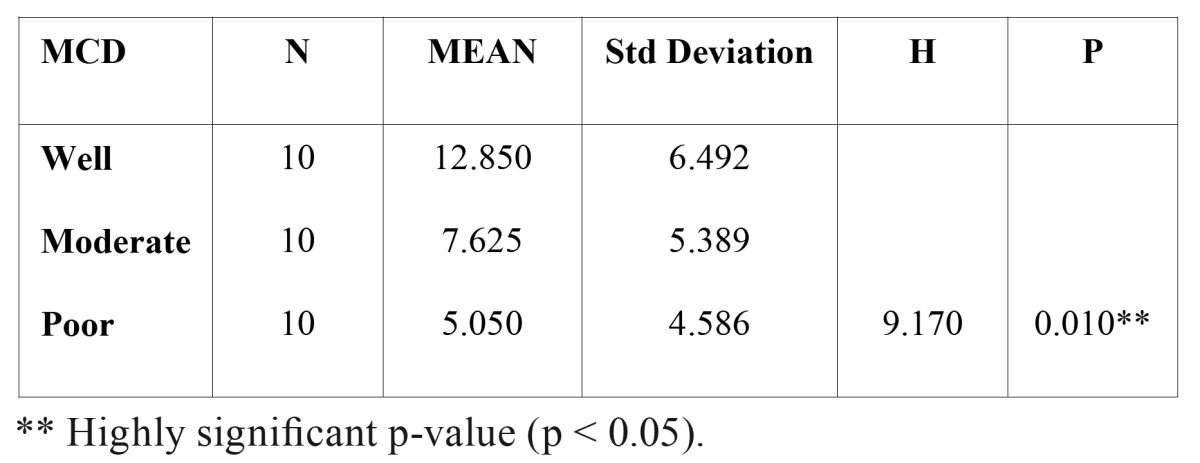
Mast cell density (MCD) in different grades of OSCC.

**Table 4:**
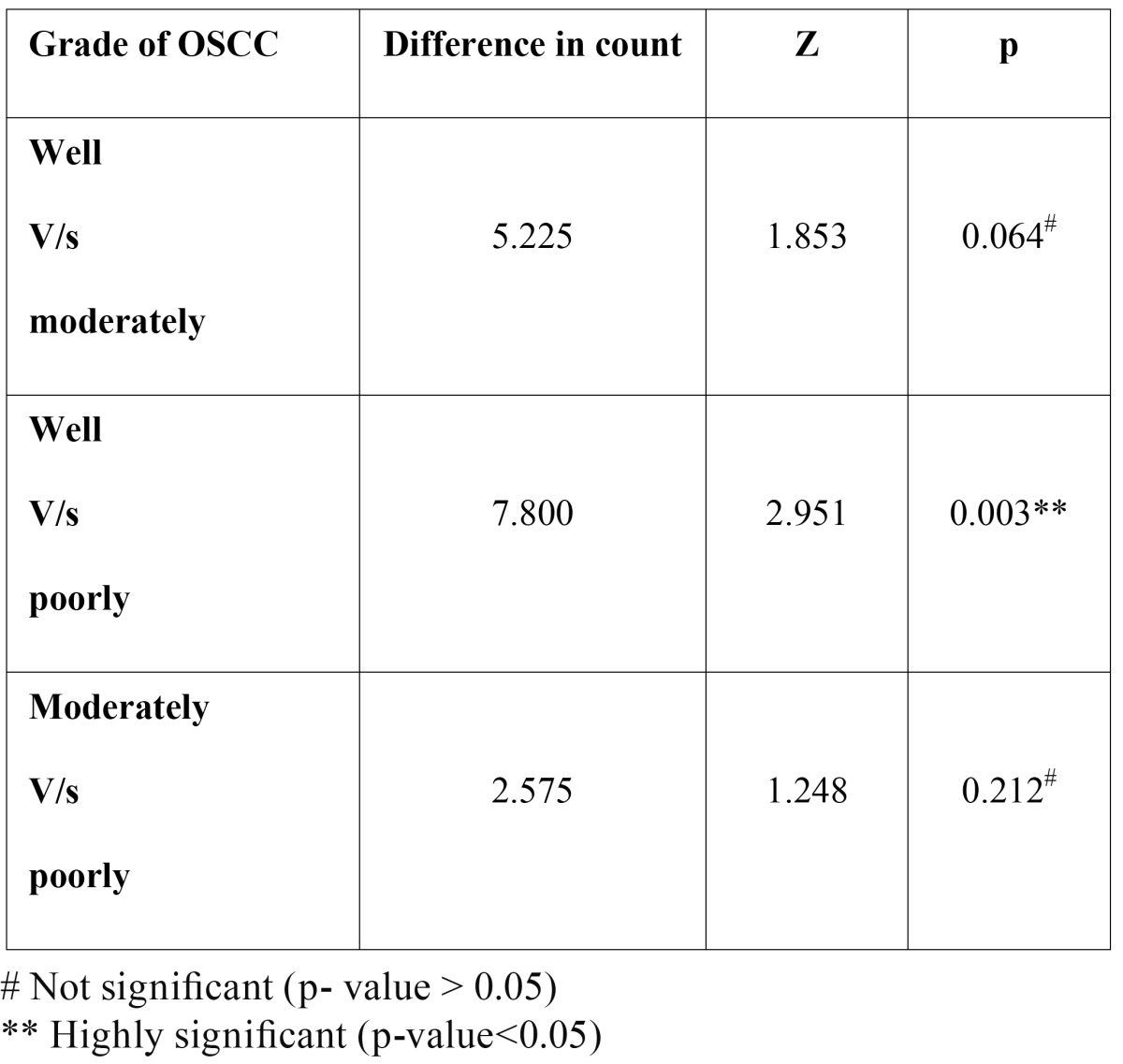
The comparison of MCD between different grades of OSCC.

## Discussion

An adequate vascular response is essential for initial development as well as continued growth of solid tumors. Experimental evidences suggest that tumor growth can be stunted by a variety of agents that have the ability to inhibit angiogenesis (5).

Currently, considerable attention has been focused on the mechanisms by which tumor acquire their blood supply for growth and dissemination. In the present study an increased angiogenesis was observed in different histological grades of OSCC with a different pattern of vascular organization. In well differentiated OSCC the vessels were arranged in a circumferential pattern located at the epithelial stromal interface; this induction of angiogenesis is probably mediated by specific angiogenic molecules released by the corresponding tumor cells, which also reflects the potentially aggressive phenotype of the tumor. Supporting the observation of Hirakawa et al. (15) on cervical carcinomas which was associated with vascular cuffing in micro vessels in cervical squamous epithelial lesions, further this pattern was related to local tumor invasion.

Studies have shown that the growth of the vessels adjacent to the tumor cells increases the opportunity for tumor cells to enter the circulation (16). Alternatively a primary tumor containing a high proportion of angiogenic cells will seed into the blood stream which will then give rise to additional metastatic deposits thus amplifying the process of growth and dissemination. This has been explained by the fact that newly proliferating capillaries have fragmented basement membranes and are leaky, making them more accessible to tumor cells than mature vessels (17). The observation made in the present study in all the 10 cases of poorly differentiated OSCC and few cases of moderately differentiated OSCC suggest a change over to a highly angiogenic phenotype to cells, as well differentiated tumor progress to poorly differentiated tumor.

Another possibility which was taken into account was the contribution of stromal inflammation. The stromal tissue in OSCC is generally rich in inflammatory reaction and focal aggregates of microvessels were seen to be associated with dense lymphoid infiltrate. The inflammatory cells are known to release angiogenic factors (18), however this view is still not clear whether they take any active part in tumor angiogenesis. Also the role of inflammatory cells in tumor stroma is controversial, as accumulation of inflammatory cells at tumor site has been considered to be host defense mechanism. On the other hand, the presence of inflammatory cells in a tumor mass represents the basis of immunosurvillence against tumor growth (19). Recent studies indicate that the inflammatory component of a developing neoplasm can provide useful means for cancer growth and metastasis mostly by potentiating extra cellular matrix remodeling and angiogenesis (20,21).

Tumor angiogenesis is a complex event mediated by angiogenic factors released from cancer cells and or by host immune cells (18,22,23). Among the host immune cells several observations have indicated the role of mast cells in tumor progression by promoting angiogenesis (22). In few malignancies large number of mast cell were detected before the occurrence of neovascularization (23). Basic FGF, IL 4, IL 8, TNF α, TNF β are among the mediators released by the granules of mast cells and are strong inducers of angiogenesis (24,25).

In the present study mast cells were counted in same areas as that of MVD in different grades of OSCC and it was found that there was a significant but inverse correlation between mast cell density and microvessel density. The number of mast cells decreased as the microvessel number increased.

These results are in contrast with the study done by Iamaroon et al. (26) in 2003 in which they found that the density of mast cells and microvessels appear to increase with the disease progression from normal oral mucosa, hyperkeratosis, premalignant dysplasia to invasive OSCC. Elpek et al. (14) in another study on squamous cell carcinoma of esophagus found a significant correlation between MVD and MCD values. Higher MVD and MCD values were also associated with tumor progression.

In the present study we found that the mast cell count decreased as the microvessel count increased with disease progression without any significant correlation between MVD and MCD. It is difficult to explain the discordant result with mast cell density in different grades of OSCC, hence it can be hypothesized that the lower density observed in all poorly differentiated OSCC is possibly due to the massive degranulation of mast cells making their identification difficult.

Reed et al. (27) found a significant increase in mast cell population surrounding melanocytic lesion expressing interleukin-3 (IL-3) compared with the lesion that did not express IL-3. In addition mast cells around these areas were found to possess IL–3 alpha receptors. They concluded that the simultaneous expression of several substances providing mast cell chemotaxis in tumor cells could influence the degree of mast cell response and could define the presence of different types of mast cells containing specific receptors against these chemoattractants. In agreement with previous studies, in the present study these differences in the type of mast cells could contribute to decrease count of mast cell during tumor progression.

It has been stated that the growth of solid tumor is dependent upon an adequate blood supply, which is achieved by generation of stroma, where the formation of new capillaries is a central event and also that this component is an entry site for immune inflammatory cells. In other words, in a particular tumor the number of microvessels and mast cells could be related to the amount of stromal component and consequently tumor having more stroma might have more mast cells and microvessels (14). For this reason variation in the amount of stroma and tumor cells in different counting fields might influence the objectivity of the microvessel counting procedure.

In the present study the MCD decreased from well to poorly differentiated OSCC. Further studies with larger sample size are needed to establish relationship between angiogenesis and mast cells.

A variety of chemotherapeutic drugs being used to treat cancer hold limited efficacy, due to problems of delivery, penetration and a moderate degree of selectivity for the tumor cells, thereby causing severe damage to healthy tissues. In addition to this, tumor cells are a rapidly changing target because of their genetic instability, heterogeneity and high rate of mutation, leading to selection and overgrowth of a drug- resistant tumor cell population (28,29).

Anti-angiogenic therapy, which targets activated endothelial cells, offers several advantages over therapy directed against tumor cells. First, endothelial cells are a genetically stable, diploid and homogenous target and spontaneous mutation rarely occur. Also, the turnover of tumor endothelial cells may be 50 times higher than that of endothelium in normal quiescent tissues, and activated blood vessels express specific markers like integrin avb3, E-selectin, Tie and VGEF receptors. Because anti-angiogenic therapy is directed at activated endothelial cells, its target should be easily accessible by systemic administration. A number of angiogenesis inhibitors are being studied; among these are endostatin, thalidomide, AE- 941(Neovastat), bevacizumab (also known as Avastin, anti-VEGF, RhuMabVEGF) and the arthritis medication celocoxib.
